# Erosive balanitis caused by *Staphylococcus haemolyticus* in a healthy, circumcised adult male

**DOI:** 10.1099/acmi.0.000582.v4

**Published:** 2023-09-08

**Authors:** José Mazuecos-Blanca, José Rafael Mazuecos-Gutiérrez, Ana Jiménez-Gil

**Affiliations:** ^1^​ Dermatology Area, Department of Medicine. Faculty of Medicine, University of Seville, Sevilla, Spain; ^2^​ Amate Health Centre, Seville District, Andalusian Health Service, Seville, Spain

**Keywords:** balanitis, balanoposthitis, circumcision, coagulase-negative staphylococci, *Staphylococcus haemolyticus*

## Abstract

**Introduction.:**

Balanitis is an inflammation of the glans penis. Balanoposthitis involves both the glans penis and prepuce and occurs only in uncircumcised males. Recurrent balanoposthitis represents a strong indication for circumcision. After *Candida* infections, aerobic bacteria are the second most common aetiological cause of acute infectious balanoposthitis, mainly streptococci groups B and D, and staphylococci, usually *S. aureus *. Their clinical manifestations are variable inflammatory changes, including diffuse erythema and oedema. Severe balanopreputial oedema with purulent exudate occurs in painful, erosive streptococcal balanoposthitis.

Coagulase-negative staphylococci (CoNS) are commensal skin bacteria, but are also recognized pathogens of the genitourinary system, mainly related to urinary tract infections. *Staphylococcus haemolyticus* is one of the main species of CoNS that is part of the cutaneous microflora but is also associated with nosocomial infections. In addition, *S. haemolyticus* also causes other infections of the male urogenital tract, such as chronic prostatitis and epididymo-orchitis, but it has not been associated with balanitis.

**Case presentation.:**

A 45-year-old man reports having suffered several episodes of balanoposthitis in the last 3 years, which were treated with topical antifungal treatments alone or associated with corticosteroids. For this reason, he underwent a postectomy by his urologist 8 months ago to avoid further recurrences.

The patient consulted for an episode of painful, erosive and exudative lesions on the glans penis and over the post-operative scars lasting 5 days. He had no urinary discomfort or inguinal lymphadenopathy. A complete blood count, biochemical analysis, C-reactive protein (CRP), prostate-specific antigen (PSA) and urinalysis were normal. Abundant growth of *S. haemolyticus* was obtained in the culture on tryptone soya agar with sheep blood and chocolate agar with Vitox media. The MicroScan panel CIM 37 (PM37) was used to study the antimicrobial susceptibilities of the isolated bacteria. The fungal culture on Sabouraud dextrose agar was negative. Based on the antimicrobial susceptibility study, treatment with oral ciprofloxacin and topical mupirocin was started, and the genital infection was completely cured.

**Conclusion.:**

We present a healthy, non-diabetic, circumcised male patient with severe, erosive and painful balanitis probably due to *S. haemolyticus *.

## Introduction

Balanitis describes inflammation of the glans penis, while balanoposthitis is an inflammation that affects both the glans penis and the foreskin [1]. They are much more common in uncircumcised males than in circumcised males, due to the occlusive effect of the prepuce, which facilitates smegma retention and inadequate hygiene [2].

Infectious balanoposthitis is the most frequent of the acute conditions. *Candida* infections are the most common cause of infectious balanoposthitis and aerobic bacteria are the second most common cause [3]. Other non-infectious causes can be traumatic, irritative or contact-based. Chronic balanitis is related to inflammatory or neoplastic dermatoses that affect the genital area.

Coagulase-negative staphylococci (CoNS) are part of the normal microbiota of the skin, but are also known pathogens of the genitourinary system, often implicated in urinary tract infections (UTIs) [4]. With respect to the pathogenicity of CoNS, there may be significant differences between species. Thus, *Staphylococcus epidermidis* and *Staphylococcus haemolyticus* have a medium pathogenic potential, while *S. carnosus* is completely apathogenic [5].

*S. haemolyticus* is one of the main species of CoNS that is part of the cutaneous microflora but is also a frequent nosocomial pathogen, mainly in immunocompromised patients or in catheter-related infections [6]. In addition, *S. haemolyticus* also causes other infections of the male urogenital tract, such as epididymo-orchitis [7] or chronic prostatitis [8], and it could be responsible for male infertility. Infection with this bacterial species decreases sperm motility and viability [6]. However, we present this clinical case because we have not found a previous association of *S. haemolyticus* with balanitis.

## Case report

A 45-year-old man reports having suffered several episodes of balanoposthitis in the last 3 years, which were treated with clotrimazole 1 % cream or miconazole 2 % and hydrocortisone 1 % cream, with only partial responses. The patient was seen in the urologist’s office, without performing blood, urine, or microbiological culture tests. Due to the persistence of these episodes, his urologist performed a postectomy 8 months ago to prevent further recurrences.

**Fig. 1.**
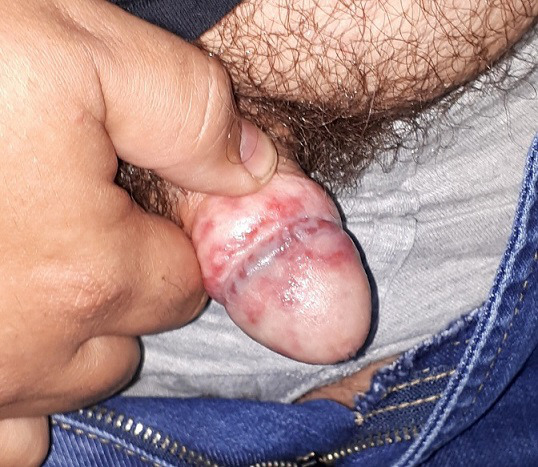
Erosions and secreting crusts on the glans penis and postectomy scar area.

However, the patient consulted for a new event of painful, erosive, and exudative lesions on the proximal half of the glans penis and over the post-operative scars of 5 days duration (Fig. 1). He had no urinary discomfort or inguinal lymphadenopathy. His wife reported no genital symptoms. A complete blood count, biochemical analysis (glucose, total cholesterol, triglycerides, urea, creatinine, total bilirubin, hepatic transaminases, sodium, potassium), C reactive protein (CRP), prostate specific antigen (PSA) and urinalysis (pH, density, glucose, protein, bilirubin, urobilinogen, ketone bodies, nitrites, red blood cells, leukocytes, microscopic examination of urinary sediment) were requested, and a sample for bacterial culture and another for mycological culture were taken from the glans lesions.

All the blood tests and urinalysis were normal. At 72 h, an abundant growth of *S. haemolyticus* was obtained in the culture on tryptone soya agar with sheep blood and chocolate agar with Vitox media incubated in 5 % CO_2_ at 25 °C. MicroScan – Positive Panel CIM 37 (PM37) was used to study the antimicrobial susceptibilities of the isolated bacteria. The results are shown in Table 1. According to the antibiogram, treatment with ciprofloxacin 500 mg every 12 h orally and with mupirocin 2 % ointment every 8 h was administered for 14 days, and the genital infection was completely cured. The fungal culture on Sabouraud dextrose agar was negative after 21 days.

**Table 1.** Antimicrobial susceptibility pattern for *Staphylococcus haemolyticus* isolated from the patient’s lesion

Susceptible	Amikacin, amoxicillin/clavulanate, cefotaxime, ciprofloxacin, daptomycin, erythromycin, gentamicin, linezolid, mupirocin, oxacillin, tetracycline, co-trimoxazole, vancomycin
Resistant	Clindamycin, penicillin, tobramycin

## Discussion

Balanitis is much more common in uncircumcised males than in circumcised males and is also more common in diabetic patients regardless of circumcision status [1]. Circumcision is therefore considered to be a surgical intervention to prevent infectious dermatoses of the penis, with or without accompanying phimosis [2]. In patients with recurrent balanoposthitis, blood glucose should be investigated for diabetes screening [1].

Yeast infections are the main causes of acute balanoposthitis, especially *Candida albicans*. The most common causes of bacterial balanoposthitis are streptococci groups B and D, and staphylococci, mainly *Staphylococcus aureus* [3]. In many studies, all patients were uncircumcised [1, 2].

The clinical significance of CoNS can be difficult to establish, as they can be either innocuous commensals or invasive pathogens. The immune status of the host will also influence the onset of CoNS disease so that the differences between pathogenic and non-pathogenic CoNS may be blurred [5]. However, our patient is a young and healthy male.

*Staphylococcus haemolyticus* is one of the cutaneous commensal CoNS. It has been observed that this staphylococcal species not only colonizes the skin, but also the urethral and periurethral surfaces of both males and females, causing urinary tract infections (UTIs) in anatomically normal men and women [6]. It has also been related to epididymo-orchitis [7] and chronic bacterial prostatitis [8]. In these reports, the single isolation of *S. haemolyticus* in microbiological culture has led to consider it as the aetiological agent.

*S. haemolyticus* is increasingly implicated in opportunistic infections, including diabetic foot ulcer infections [4]. Severe infections such as otitis, endocarditis, prosthetic joint infections, meningitis, peritonitis and bacteraemia have been reported, especially in immunocompromised patients. The presence of venous catheters or medical devices increases the risk of infections due to the ability of *S. haemolyticus* to form biofilms [6].

*S. haemolyticus *, especially strains that cause nosocomial infections, are more resistant to antimicrobial drugs than other CoNS and could be an emerging threat today. It seems that the most important factor could be the ability to acquire multidrug resistance to various antibiotics, including glycopeptides [9]. However, in our case there was a good response to the antimicrobial treatment administered.

Although *S. haemolyticus* is one of the most frequently encountered aetiological agents for staphylococcal infections [4], we have not found a previously recorded relationship with balanitis. There is also no mention of its involvement in the European guidelines for the management of balanoposthitis. These indicate that streptococci and *S. aureus* are the aerobic bacteria that cause balanitis, although others may be involved, but the species are not specified. This paper reports that the clinical manifestations are variable inflammatory changes, including diffuse erythema and oedema, and that treatment is usually topical, while severe cases may require systemic antibiotics, depending on the susceptibilities of the isolated micro-organism [3]. In erosive and painful streptococcal balanoposthitis, there may be severe balanopreputial oedema with purulent exudate [10]. However, our patient’s circumcision should hinder the development of balanitis.

In the study by Deepa *et al*., *S. aureus* and *Staphylococcus epidermidis* were the most frequently isolated bacteria in patients with balanoposthitis [11]. Another CoNS, *Staphylococcus warneri *, has also been associated with balanoposthitis and correlated positively with disease severity [12]. In both studies, all patients were uncircumcised.

A case of epididymo-orchitis associated with *S. haemolyticus* bacteraemia in a healthy adult male has been described [7]. This urogenital condition could be due to bacteria found in the urogenital or gastrointestinal tract of a healthy female partner, through vaginal or anal intercourse, causing infection of the urinary bladder or urethra, refluxing to the epididymis and then to the testis. In our case, these bacteria may have only infected the patient’s external genitalia.

We believe that *S. haemolyticus* is the causative agent of the patient’s infection; however, the diagnostic evidence is not definitive and may present some limitations, since the infection was caused by a commensal skin staphylococcus isolated from a skin smear taken from a non-sterile area. In addition, the treatment administered was broad spectrum and could cover various Gram-positive and Gram-negative bacteria.

In conclusion, we present a case with severe, erosive and painful balanitis, probably due to *S. haemolyticus* in a healthy, non-diabetic, circumcised male. To our knowledge, this could be the first reported case of balanitis due to *S. haemolyticus *.
